# Editorial: Implementation of genomic medicine in Africa: One continent, one vision

**DOI:** 10.3389/fgene.2022.1133118

**Published:** 2023-01-10

**Authors:** Maritha J. Kotze, Tivani P. Mashamba-Thompson, Dawn Stephens

**Affiliations:** ^1^ Division of Chemical Pathology, Department of Pathology, Faculty of Medicine and Health Sciences, Stellenbosch University and National Health Laboratory Service, Tygerberg Hospital, Cape Town, South Africa; ^2^ Faculty of Health Sciences, University of Pretoria, Pretoria, South Africa; ^3^ Technology Innovation Agency, Pretoria, South Africa

**Keywords:** pathology, personalised medicine, precision medicine, policy, translational research, genomics platform, data, african genomes

The translation of genetic research from bench to bedside involves multiple choices related to clinician-patient shared decision-making, with ethical implications at every step of the way. Given the evolving evidence base and limited genomic data from African genomes, we were delighted to receive three reviews, one opinion article, three perspectives, six original papers, and a brief research report with a checklist for assessment of the readiness to implement public health genomics. The latter contribution by Jongeneel et al. included survey results previously generated in parallel to the development of a framework for implementation of genomic medicine in Africa, which was published in February 2021 on commission of the African Academy of Sciences (https://www.aasciences.africa/publications/policy-paper-framework-implementation-genomic-medicine-public-health-africa). The Policy Brief summarized this framework for personalized genomic medicine as the foundation of the current translational Research Topic, showcasing collated evidence of applied knowledge in Africa to enable translation of research into clinical practice, as the study endpoint. The wide range of methodologies used, and implementation approaches presented, were evaluated for evidence of transition from population to individualised risk stratification required for the application of personalised genomic medicine.

In the first review submitted, Govender et al. highlighted the need for development of preventive intervention strategies that would delay the onset and slow progression of chronic kidney disease through treating the underlying aetiologies in at-risk individuals. Screening and early detection of disease pathways utilizing biomarkers at critical control points in pharmacogenetic algorithms were recommended for future implementation of targeted therapy and preventative interventions. Zondo et al. furthermore urged the need to better understand the mechanisms of drug availability and metabolism of antiretrovirals, as the effectiveness of therapy depends on adequate drug delivery and availability to sites of HIV infection. Genetic polymorphisms and genital inflammation may influence drug transport and lead to contradictory results in clinical trials, posing a significant implementation barrier that needs to be addressed by pharmacogenetic studies in genetically diverse populations to improve the efficacy of drug dosing and delivery. In Egypt the Reference Genome Project has initialized the era of personalised medicine as reported by El-Attar et al. These authors highlighted the challenges and first steps taken to fulfil the promise of the “right drug administered to the right patient at the right time”. The link between serum 25 (OH) Vitamin D level and breast cancer prognosis was used as an example of where the pathology-supported genetic testing framework implemented as a case study in South Africa may be useful to overcome limitations, given the effect on musculoskeletal health.

In a summary of the current state of esophageal squamous cell carcinoma (ESCC) genomic research in Africa, Simba et al. expressed the opinion that implementation of genomic medicine for ESCC remains elusive due to a lack of multi-omics studies in African populations. The finding that several mutational signatures in ESCC have been linked to environmental exposures necessitates the incorporation of known environmental and lifestyle risk factors when screening for genetic factors. No standardized methods for data analysis and reporting exist to allow the implementation of a targeted multi-disciplinary intervention plan. Small sample sizes and omission of controlling for population substructure in admixed populations are major limitations together with the complexities associated with data sharing, transfer and storage. Similar perspectives were shared by Ghoorah et al. from the Southwest Indian Ocean region that is in urgent need of evidence from local genetic data to assess the benefits of implementing genomics in healthcare. While well-established genetic testing of the major cancer susceptibility genes, *BRCA1* and *BRCA2*, is not yet available in the public sector, transcriptional gene profiling (70-gene MammaPrint test) has been introduced in the private sector following medical scheme reimbursement in Mauritius to prevent chemotherapy overtreatment in patients with early-stage breast cancer. Hurrell et al. provided the rationale for cellular hepatic models as a useful tool to validate African relevant gene-drug interactions. A convincing use case was presented for researchers to improve the transferability of global research findings to an African relevant context. In a further perspective from the same research group, Nkera-Gutabar et al. make a strong argument in favour of the human microbiome as a valuable complementary approach to traditional genomic medicine. Less than 2% of microbiome diversity is explained by host genetics, while environmental factors associated with diet and lifestyle account for ∼20% of gut microbiome variance. Rapid transition to an increasingly westernized environment over the last 2 decades has reshaped the human microbiome in parallel with an increased prevalence of non-communicable diseases (NCDs) such as obesity, neurological disorders and cancer.

Translation of the research findings of two cross-sectional microRNA-related epigenetic studies performed in South African patients with type 2 diabetes mellitus (Weale et al.) and hypertension (Matshazi et al.), is hampered by a lack of clinical data on complications pertaining to these NCDs. This led to recommendations to clarify the effect of non-coding RNAs by building on the current knowledge base and expertise developed within the research team. As a result of this project, it may be possible in future to develop a panel of biomarker targets for cardiometabolic risk stratification offering potentially novel prognostic and therapeutic avenues at the individual level. A retrospective study reported by Tshabalala et al. provided a unique and large HLA dataset of South Africans, which may be a useful resource for future research despite significant limitations imposed by data missingness, imbalance of sample sizes and methodological difficulties. In the cross-sectional study from Kenya, Gatua et al. described the cytogenetic and molecular abnormalities of acute myeloid leukemia in ten patients treated at the hemato-oncology unit of Kenyatta National Hospital over a 3-month period. Comparison between targeted next-generation sequencing (NGS) and whole exome sequencing (WES) after variant filtering for African ancestry, showed high concordance with many variants of uncertain clinical significance detected by both methods. Although exon capturing did not cover all regions of interest, WES allowed for detection of clinically relevant variants implicated in drug resistance and comorbidities such as hypercholesterolemia as an added benefit when integrating research and service delivery. Van der Merwe et al. and Van der Merwe et al. reported on targeted *BRCA1/2* gene screening available in the public sector of South Africa since 1998, and stakeholders’ views on broad-spectrum WES first used as a discovery tool in the private sector in South Africa, respectively. Both research groups expressed their support for future implementation of first-tier point-of-care screening and online genetic counselling platforms. This will increase access to appropriate genetic testing and with addition of WES in uninformative cases, offer the potential to better differentiate between inherited, lifestyle-triggered and therapy-associated disease manifestation. Low uptake of *BRCA/*other cost-effective genomic tests despite proven clinical utility is concerning.

Implementation of genomic medicine in Africa will remain an elusive goal unless the researchers who best understand the benefits and limitations of their results take action to make personalised genomic medicine a reality. The Open Genome project is just one of many initiatives acting out the insights gained through developing and applying the readiness checklist published by Jongeneel et al. This research translation tool listing eight requirements for implementation of genomic medicine programs in Africa, proved useful to evaluate the practicalities of recommendations made in 1) the *review papers* to bridge existing clinical implementation gaps identified, 2) to assess examples of knowledge application based on previous experiences reflected in the *opinion piece* and *perspectives*, and 3) to screen *original articles* for evidence of inventive steps taken or recommended to spearhead the implementation of genomic healthcare solutions in Africa and beyond. Deep knowledge gained during these studies should be translated responsibly without delay into practical applications that will benefit study participants and the population at large.

We echo the idea set forward by Nkera-Gutabara et al. to position the microbiome as the “second genome”, giving researchers a second chance to prioritise the development of technologies and datasets that drive the African health agenda together (pamoja!) in a way that embodies the “One Continent: One vision” ethos of this Research Topic.

